# Supplementary material for: Pooled prevalence and its determinants of stunting among children during their critical period in Ethiopia: A systematic review and meta-analysis
Source: PLoS One. 2023 Nov 29;18(11):e0294689. doi: 10.1371/journal.pone.0294689 (PMC10686443; doi:10.1371/journal.pone.0294689)
Supplement: S1 Table — (DOCX) [file pone.0294689.s001.docx]

| S1 table: Studies search strategies and entry terms from different electronic data bases on the prevalence and determinants of stunting less than 24 months children in Ethiopia | |
| --- | --- |
| Search in each key words and Mesh terms | **Pumed/MEDLINE database** |
| **#1** | "Prevalence"[Mesh] OR prevalence[tw] OR proportion [tw] OR burden [tw] OR magnitude [tw] OR epidemiology[tw] |
| **#2** | “associated factor*” [tw] OR "Risk Factor*"[Mesh] OR determinants[tw] OR "risk factor*"[tw] OR etiology[tw] OR predictors[tw] |
| **#3** | Stunting[tw] OR “chronic malnutrition”[tw] OR “chronic undernutrition” [tw] OR undernutrition[tw] OR "Nutritional Status"[Mesh] OR nutritional status[tw] |
| **#4** | “children 6-23 months”[tw] OR “under two years ”[tw] OR “infants and young child*”[tw] OR “children less than 2 years”[tw] OR” young child*”[tw] OR “children 6-24 months”[tw] |
| **#5** | Ethiopia |
| **Final** | "Prevalence"[Mesh] OR prevalence[tw] OR proportion [tw] OR burden [tw] OR magnitude [tw] OR epidemiology[tw] AND “associated factor*” [tw] OR "Risk Factor*"[Mesh] OR determinants[tw] OR "risk factor*"[tw] OR etiology[tw] OR predictors[tw] AND Stunting[tw] OR “chronic malnutrition”[tw] OR “chronic undernutrition” [tw] OR undernutrition[tw] OR "Nutritional Status"[Mesh] OR nutritional status[tw] AND “children 6-23 months”[tw] OR “under two years ”[tw] OR “infants and young child*”[tw] OR “children less than 2 years”[tw] OR” young child*”[tw] OR “children 6-24 months”[tw] AND Ethiopia |
| **Search results** | **97** |
| **Search date** | **March/12/2023** |
|  | **Google scholar database** |
| With all of the words | (Prevalence AND determinant AND stunting children AND “6-23 months") AND Ethiopia |
| With exact phrases | “Children 6-23 months” |
| with **at least one** of the words | ("Risk factors" OR "associated factors" OR "chronic malnutrition" OR "under 2 years" OR "children "6-24 months" "6-23 months") which yields =753 any type of article |
| **Final Search builds** | (Prevalence AND determinant AND stunting children AND “6-23 months") AND Ethiopia AND ("Risk factors" OR "associated factors" OR "chronic malnutrition" OR "under 2 years" OR "children "6-24 months" "6-23 months") |
| **Total search results** | **753** |
| **Search date** | **March/14/2023** |
