# Supplementary figures and images for: Pooled prevalence and its determinants of stunting among children during their critical period in Ethiopia: A systematic review and meta-analysis
Source: PLoS One. 2023 Nov 29;18(11):e0294689. doi: 10.1371/journal.pone.0294689 (PMC10686443; doi:10.1371/journal.pone.0294689)

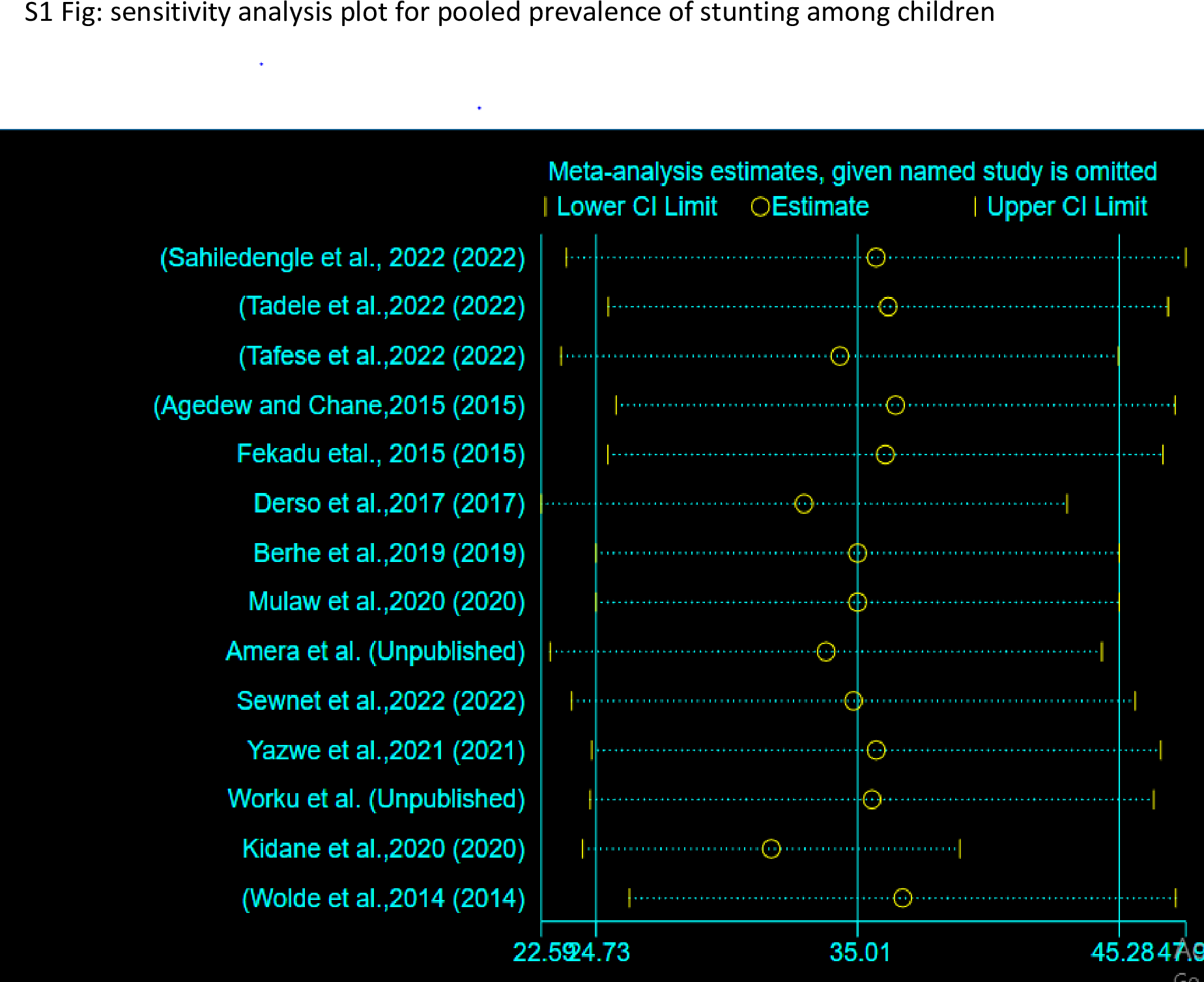

Supplement: S1 Fig — (TIF) [file pone.0294689.s002.tif]

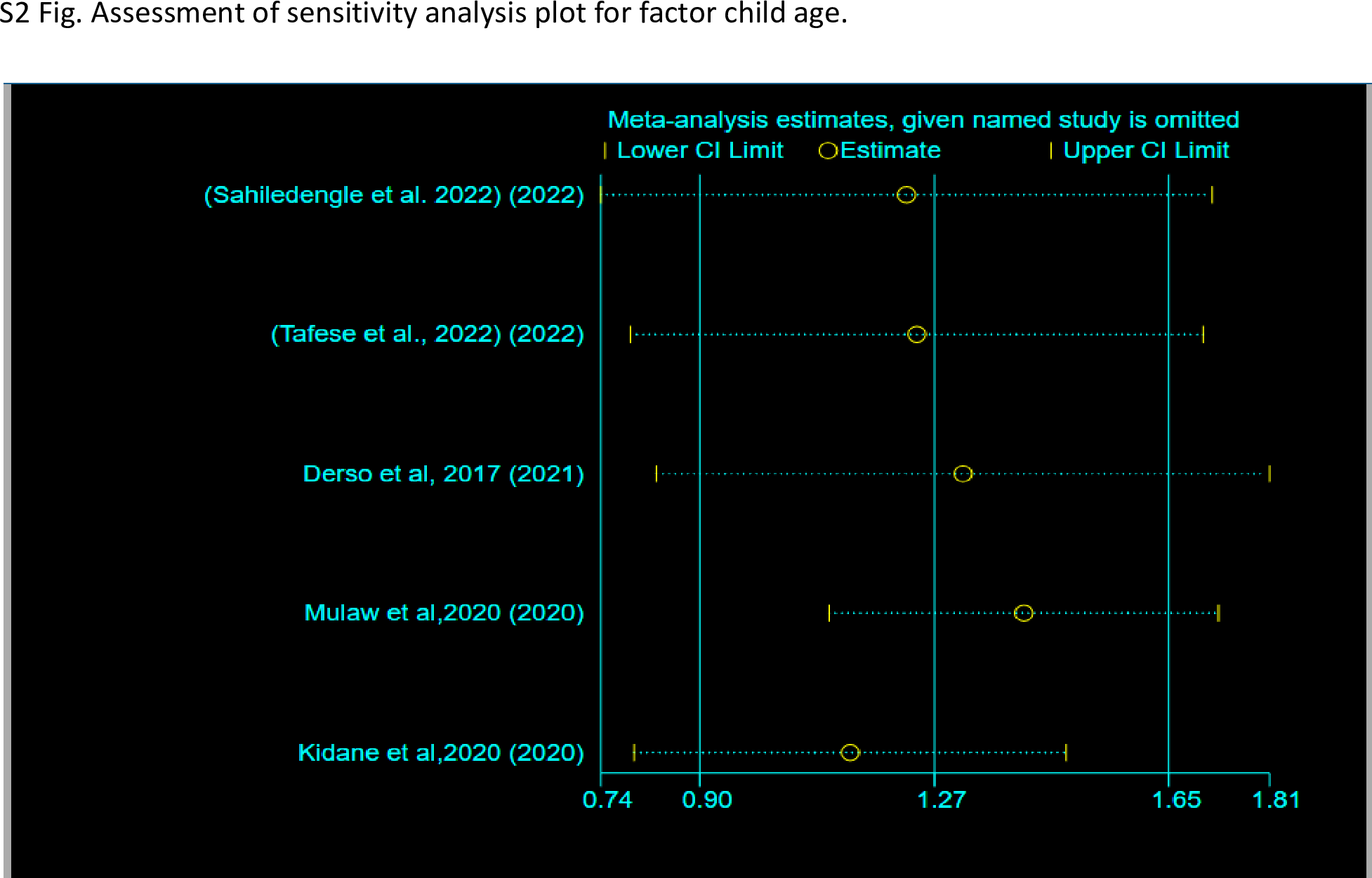

Supplement: S2 Fig — (TIF) [file pone.0294689.s003.tif]

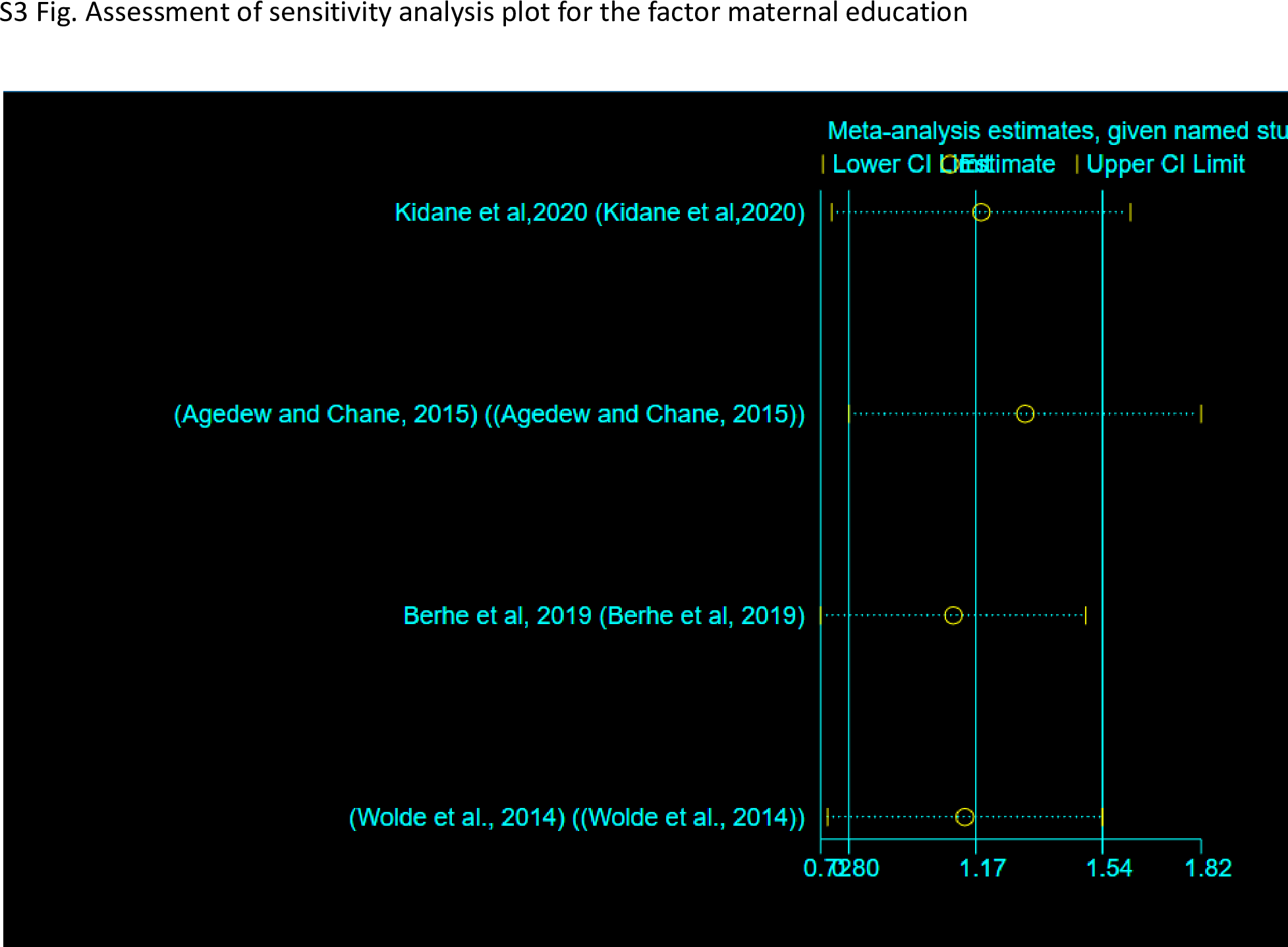

Supplement: S3 Fig — (TIF) [file pone.0294689.s004.tif]

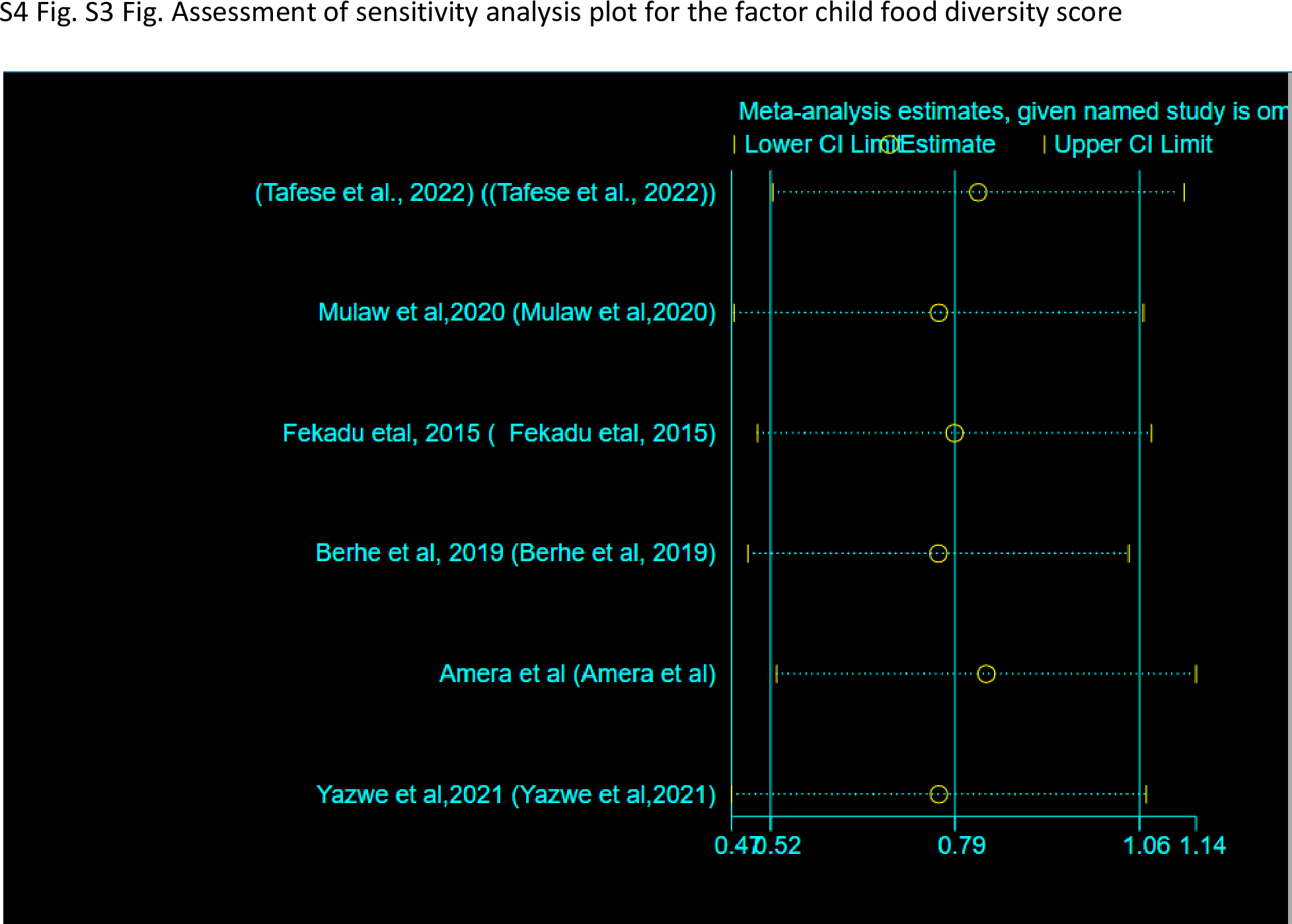

Supplement: S4 Fig — (TIF) [file pone.0294689.s005.tif]
