# Supplementary material for: Pooled prevalence and its determinants of stunting among children during their critical period in Ethiopia: A systematic review and meta-analysis
Source: PLoS One. 2023 Nov 29;18(11):e0294689. doi: 10.1371/journal.pone.0294689 (PMC10686443; doi:10.1371/journal.pone.0294689)
Supplement: S2 Checklist — (DOCX) [file pone.0294689.s007.docx]

**JBI’s critical appraisal Checklist for cross-sectional studies**

| Primary studies | JBI’s critical appraisal questions | | | | | | | | Overall quality score (%) | Included |
| --- | --- | --- | --- | --- | --- | --- | --- | --- | --- | --- |
|  | Q1 | Q2 | Q3 | Q4 | Q5 | Q6 | Q7 | Q8 |  |  |
| (Sahiledengle et al., 2022) | Y | Y | Y | Y | Y | N | Y | Y | 87.5% | √ |
| (Tadele et al., 2022) | Y | Y | Y | Y | N | N | Y | Y | 75% | √ |
| (Tafese et al., 2022) | Y | Y | Y | Y | Y | N | Y | Y | 87.5% | √ |
| (Agedew and Chane, 2015) | N | Y | Y | Y | Y | N | Y | Y | 75% | √ |
| (Derso et al., 2017) | N | Y | Y | Y | Y | N | Y | Y | 75% | √ |
| (Wolde et al., 2014) | N | Y | Y | Y |  | N | Y | Y | 75% | √ |
| Amera et al. | Y | Y | Y | Y | Y | N | N | N | 87.5% | √ |
| (Sewenet et al., 2022) | Y | Y | Y | Y | Y | N | Y |  | 87.5% | √ |
| (YAZEW and BEKELE, 2021) | Y | Y | Y |  | Y | N | Y | Y | 87.5% | √ |
| Worku et al. | Y | Y | Y |  | Y | N | Y | Y | 87.5% | √ |
| (Kidane et al., 2020) | N | Y | Y | Y | Y | N | Y | Y | 75% | √ |
| (Fekadu et al., 2015) | N | Y | Y | Y | Y | N | Y | Y | 75% |  |

| Q1. Were the criteria for inclusion in the sample clearly defined? |  |  |  |  |
| --- | --- | --- | --- | --- |
| Q2. Were the study subjects and the setting described in detail? |  |  |  |  |
| Q3. Was the exposure measured in a valid and reliable way? |  |  |  |  |
| Q4. Were objective, standard criteria used for measurement of the condition? |  |  |  |  |
| Q5. Were confounding factors identified? |  |  |  |  |
| Q6. Were strategies to deal with confounding factors stated? |  |  |  |  |
| Q7. Were the outcomes measured in a valid and reliable way? |  |  |  |  |
| Q8. Was appropriate statistical analysis used? |  |  |  |  |

**NB**. Y: Yes, N: No, U: Unclear, Q: Question. The overall score is calculated by counting the number of Y’s in each row.

JBI’s critical appraisal checklist for case control studies

| **Primary studies** |  | **JBI’s critical appraisal questions** | | | | | | | | | Overall quality score (%) | Included |
| --- | --- | --- | --- | --- | --- | --- | --- | --- | --- | --- | --- | --- |
|  | Q1 | Q2 | Q3 | Q4 | Q5 | Q6 | Q7 | Q8 | Q9 | Q10 |  |  |
| Berhe et al.,2019 | Y | Y | Y | Y | Y | Y | Y | Y | N | Y | 90% | √ |
| Mulaw et al.,2020 | Y | Y | Y | Y | Y | N | Y | Y | N | Y | 80% | √ |

Q1: Were the groups comparable other than the presence of disease in cases or the absence of disease in controls?

Q2: Were cases and controls matched appropriately?

Q3: Were the same criteria used for identification of cases and controls?

Q4: Was exposure measured in a standard, valid and reliable way?

Q5: Was exposure measured in the same way for cases and controls?

Q6: Were confounding factors identified?

Q7: Were strategies to deal with confounding factors stated?

Q8: Were outcomes assessed in a standard, valid and reliable way for cases and controls?

Q9: Was the exposure period of interest long enough to be meaningful?

Q10: Was appropriate statistical analysis used?

**NB**: Y: Yes, N: No, U: Unclear, Q: Question. The overall score is calculated by counting the number of Y’s in each row.
