# Supplementary material for: Pooled prevalence and its determinants of stunting among children during their critical period in Ethiopia: A systematic review and meta-analysis
Source: PLoS One. 2023 Nov 29;18(11):e0294689. doi: 10.1371/journal.pone.0294689 (PMC10686443; doi:10.1371/journal.pone.0294689)
Supplement: Attachment — Submitted filename: Stunting.docx [file pone.0294689.s008.docx]

**Manuscript Number:** PONE-D-23-17150

**Manuscript Title:** Pooled prevalence and its determinants of stunting among children during their critical period in Ethiopia: A systematic review and Meta-analysis.

Congratulations dear authors on your scholarly work based on a priori protocol registered in PROSPERO; you have brought an important study problem with good findings that have public health importance in optimizing children’s health. However, there few methodological issues that I want you to address before considering the manuscript for publication.

**General comment**

There are several typological and grammar usage errors that need extensive proof reading for revisions.

**Specific comments**

**Methods**

I suggest the authors consider clear explanation of their PICO mnemonic to so that it will be easier for the readers. Just describe each component separately.

How did the authors look for grey literature?

Kindly append a table showing methodological quality of the appraised articles with the last column being ‘overall quality score’.

Explain if data transformation was required or undertaken when data were reported differently.

**Results**

Please, use PRISMA 2020 flow diagram. <https://prisma-statement.org/PRISMAStatement/FlowDiagram>

Please include two columns in Table 1: data collection technique (interview, observation, self administered questionnaire, etc) and funding source for each study (You can say not funded, not reported or name of funder if funded).

Good luck!!
