# Supplementary material for: Pooled prevalence and its determinants of stunting among children during their critical period in Ethiopia: A systematic review and meta-analysis
Source: PLoS One. 2023 Nov 29;18(11):e0294689. doi: 10.1371/journal.pone.0294689 (PMC10686443; doi:10.1371/journal.pone.0294689)
Supplement: Attachment — Submitted filename: Response to Reviewers.docx [file pone.0294689.s010.docx]

**Response to Reviewers**

**Point by point responses**

We would like to take this opportunity to thank the reviewers and editor for sharing their view and constructive comments. The comments were very important which further improves the quality of the manuscript. The point-by-point responses for each of the comments are provided in the following pages. **Our responses are written in blue font color.**

**# Editor Comment**

1. Please ensure that your manuscript meets PLOS ONE's style requirements, including those for file naming

**Authors’ Response**

We are grateful to this comment of technical relevance. Thus, we have ensured that our manuscript meets PLOS ONE's style requirements, including those for file naming.

2. We noticed you have some minor occurrence of overlapping text with the following previous publication(s), which needs to be addressed.

**Authors’ Response**

**Thank you**. The authors have checked carefully the overlapping texts and paraphrased the duplicating works on the revised document.

3. Please review your reference list to ensure that it is complete and correct. If you have cited papers that have been retracted, please include the rationale for doing so in the manuscript text, or remove these references and replace them with relevant current references. Any changes to the reference list should be mentioned in the rebuttal letter that accompanies your revised manuscript. If you need to cite a retracted article, indicate the article’s retracted status in the reference list and also include a citation and full reference for retraction notice.

**Authors’ Response**

The authors are very grateful of these constructive comments. After read the reference lists carefully, we made a correction and addressed the incomplete one. The authors also have noticed the retracted articles on the reference list and removed these references and replace with relevant current references. But the authors did not removed the reference 39 and 40 since the source is book in which it has an updated version.

Reviewer one

1. There are several typological and grammar usage errors that need extensive proof reading for revisions.

**Authors’ Response**

First of all, we thank you the reviewer for his constructive comments. After we have read carefully through the whole document, we properly addressed the concerned issues.

Accepting the comment, the authors have read thoroughly and edited carefully the whole manuscript before submission.

2. I suggest the authors consider clear explanation of their PICO mnemonic so that it will be easier for the readers. Just describe each component separately.

**Authors’ Response**

Thank you for your insight; accepting the comment, we made a clear explanation of PICO mnemonic on the revised version of the manuscript (page 5).

3. How did the authors look for grey literature?

**Authors’ Response**

The authors have dealt on the issue of including grey literature. After deep discussion, we decided to include two articles based on the following reasons:

- Based on the quality assessment, using critical appraisal checklists (JBI), these articles have low risk.
- The authors consider that they had been assessed and evaluate since these articles were found from universities repositories.
- Some evidences have recommended to consider these literatures if meeting certain criteria.

4. Kindly append a table showing methodological quality of the appraised articles with the last column being ‘overall quality score’.

**Authors’ Response**

We accepted the comment and correct on the revised manuscript. Moreover, the methodological quality of the appraised articles were presented in detail in supplementary checklist two (S2 checklist) as cited on page 6.

5. Explain if data transformation was required or undertaken when data were reported differently.

**Authors’ Response**

Thank you for your concern. The authors did not undertake data transformation since it was not required. The data of all the included studies were presented similarly (log odds).

6. Please, use PRISMA 2020 flow diagram

**Authors’ Response**

Accepting the comment, the authors made a revision on the revised document.

7. Please include two columns in Table 1: data collection technique (interview, observation, self-administered questionnaire, etc.) and funding source for each study (You can say not funded, not reported or name of funder if funded).

**Authors’ Response**

Accepting the comment, we made a correction on the revised version of the manuscript (page 7-9).
